# Some Remarks on Scurvy

**Published:** 1848-06

**Authors:** R. S. Holmes

**Affiliations:** St. Louis, late of the United States Army


### RECORD OF MEDICAL SCIENCE.

*Some Remarks on Scurvy.* By R. S. Holmes, M. D., *of St. Louis, late of the United States Army.*—My attention was particularly called to this disease, by many well-marked, yet rather anomalous cases of it, that I witnessed with the army in Florida. I have seen it elsewhere since then, throughout the United States, and in Mexico, and I am convinced it is often overlooked or not suspected; that the names of other diseases had been given to it, and that from its diversified character, it defies all attempts at a complete history of its symptoms. The only well-marked proof that many diseases I have seen, have been affected by the scorbutic constitution of the fluids, has been in the *cure;* if an inflamed eye, or an ulcerated leg, is cured by a drink of acids and a diet of vegetables, when the patient, for some time previously, has been living on salt provisions and without vegetables, it affords presumptive proof that the disease is of a scorbutic origin.

Scurvy stamps its impress on diseases very much in the manner that malaria is in the habit of doing. There is no disease that is not capable of taking on a scorbutic character—as far as I have had an opportunity of judging—even although the accredited symptoms of scurvy itself may not be present: nay, I believe there is scarcely an individual among the many who escape when exposed to scorbutic causes, who has not within him the *leaven* of the disease, capable of giving signs of its presence, if not by absolute symptoms, still by the stamp of the scorbutic diathesis on any other disease to which the system may be liable.

It is a great error to suppose that in all cases of scurvy the gums are affected, or that the patient is depressed in spirits, or that he has a peculiarly sallow look, or that he loses his strength, or has livid colored patches, as if of extravasated blood beneath the skin; these may all be present, or not one of them, and yet the patient will have scurvy. How then, it may be asked, do you recognize the disease? We answer, by the certain causes that are known to produce it, and by the readiness with which acids or vegetables act favourably. We have certainly seen no single symptom present in all cases of scurvy; probably the most common signs of the disease are the patches, as if of purpura, which are seen beneath the skin, of an unhealthy liver colored appearance; these most generally occur on the arms, then on the legs and chest, or ankles, or some part of the face, but scarcely ever on any other part of the body. There is generally a puffiness of the gums, or they become tender and bleed, or the teeth become loose; but a very universal sign of the disease in Florida, was the superficial extended ulcerations, or sheets of bullæ, giving rise afterwards to suppuration, which appeared on the feet and ankles, and between the toes. This inflammation is very peculiar and characteristic: I think I have seen it in one half, of probably one hundred cases of scurvy, that I have treated; it originates by a crop of small vesicles, similar to those raised by a cantharides blister, sometimes remaining distinct, and often running into each other; these burst, and leave underneath a raw, partially suppurating, surface, of probably the size of a dollar, scattered here and there over the foot or ankle, or between the toes. The first cases I saw of this kind, were in the hospital at Tampa Bay. With a command of four hundred men, I had generally from thirty to fifty on the sick report. I had probably treated a dozen of these cases of inflammation, before I suspected the cause of disease—with what success, may be imagined, when the diagnosis was incorrect. I have had patients for three months on the sick report, with this inflammation, when the cure was probably brought about at the end of that time, by the anti-scorbutic diet that was habitually used in the hospital, as far, at least, as circumstances would permit: yet afterwards I was in the habit of curing these inflammations in a few days' time, by drinks of lemonade, or, what is better, I think, by a mixture of vinegar and the nitrate of potash, in as large doses as the stomach will bear; and by a diet of potatoes, and, if you wish, salt beef or pork, also; for it is not the *presence* of these articles, but the *absence* of acid vegetables that produces scurvy: sourkrout, too, the "unfermented cabbage of the Dutch," is a good article of diet, and can be had and preserved where potatoes cannot—but it is difficult for the stomach to digest it well, and is not equal to potatoes as an anti-scorbutic diet.

Another one of the most common forms, in which scurvy betrayed itself, is in inflammation of the conjunctiva, or lids of the eye. I am not certain that this disease was of a scorbutic origin, but certainly no sooner would such an inflammation set in, than it was seized upon and overshadowed by the scorbutic diathesis; and I am much inclined to believe, that this inflammation was caused by the seeds of scurvy in the system. Ulceration of the cornea was also another very common form of disease of the eye, in which this diathesis played the chief part. In examining a recruit last winter, at Fort Snelling, near the Falls of St. Anthony, a very stout man, well formed, and apparently in good health, I was struck with the appearance of three ulcers on the cornea of one eye; the other was not strong, but very little inflamed. This has generally been with me a cause of rejection—for the disease betokens a constitutional or organic fault somewhere—but I concluded to pass this man, as he was otherwise stout and able-bodied, and it was not necessary that he should do duty immediately. I took him into the hospital and treated him by all the known modes I could think of, for the cure of ulceration of the cornea; first by touching the ulcers with nitrate of silver, by antiphlogistics, with absence of light and confinement to the house—then by stimulants and exercise out of doors; but all was of no avail; at the end of two months the eye was as much diseased as when he first entered the hospital. I enquired into his former habits of life, and found he had been a sailor, but had been for some months working in the lead mines of Wisconsin, and almost wholly deprived of vegetable diet. I suspected the cause—

put the patient upon a strict anti-scorbutic diet, and by occasionally touching the ulcers with nitrate of silver, healed them in two weeks' time. This case brought to my recollection another peculiar feature of scurvy that I had often seen verified in Florida; that is, the facility with which relapses take place. This man troubled me twice again during the winter and spring with the same disease, which I cured in the same manner; for the supply of potatoes for the men at the Fort was small, and they were of a bad quality, the acid of the potato being probably destroyed by the severe cold and age. I may incidentally mention, for the benefit of those who examine recruits, that this was one of three or four cases that I have passed where the cornea was ulcerated, and in this, as in every other case, I have had cause to regret it. I think it ought always to be placed among the disqualifying circumstances.

Soldiers subject to the phlegmasiæ, and also to a scorbutic diathesis, were among our most frequent patients in Florida; the relapses were so frequent, that such men were scarcely ever out of the hospital. By the aid of a proper diet, we could easily accomplish a cure for the time being, but the disease would return in a week or ten days after the diet, peculiar to troops when in that state, was used; ulcers on the legs, burrowing inflammations after wounds, the occurrence of a very foul and unhealthy state of the gums after the extraction of a tooth, and a peculiar susceptibility to salivation from the use of mercury, or other medicinal agents, were among the most common effects of the scorbutic constitution; added to these were those signs recognised most generally as indicative of scurvy—tender gums, liver colored blotches, sallow countenance, depressed spirits, (probably of all the most common symptoms,) secretions altered, bowels deranged, and muscular system weakened.

I wish to call the attention of some of your readers to this disease, for I am convinced it is a much more common one in the country than is generally supposed. It is a great error to believe that, because one lives out of a town, that fresh vegetables can be procured; the country, as far as my experience goes, is often the most difficult place to get them. Potatoes could never be had at most of our frontier posts, were they not cultivated by the soldiers themselves, and preserved during the winter; and it is quite a source of profit to the soldiers to sell their extra supply to the country people around, for their own consumption; they are the great prophylactics in scurvy, and generally the only vegetable used during nine months of the year, in the northern part of the United States. But the supply in new settlements is often very limited, while in the lumber regions of Maine, New York, on the head waters of the Mississippi, and in the mining districts of Illinois, Wisconsin, and Iowa, the workmen are deprived of any vegetables whatever for many months of the year; their diet consists of pork, beans, hard bread, and coffee. No conception can be formed, by any one who has not been much in the untrodden ways of the country, of the extent to which salt pork, as an article of diet, is used; not that *it* produces scurvy, but the facts are, that where it is much used, vegetables are not, for the simple reason that they cannot be procured. Moisture, one of the greatest excitors of the disease, is wanting, in a great measure, in the interior of the country, and this is one cause of the disease being circumscribed. I have observed that it is more prevalent along the sea coast, and this is, probably, one of the chief reasons of its frequent occurrence on the ocean. —*St. Louis Med. & Surg. Jour.*

